# Purinergic P2X receptor 7 (P2X7R) inhibition induced cytotoxicity in glioblastoma

**DOI:** 10.1371/journal.pone.0332212

**Published:** 2025-09-16

**Authors:** Matthew Drill, Liyen Katrina Kan, Richard P. Sequeira, Padmakrishnan Chorakode Jayakrishnan, Martin Hunn, Nigel C. Jones, Terence J. O’Brien, Mastura Monif

**Affiliations:** 1 Department of Neuroscience, School of Translational Medicine, Faculty of Medicine, Nursing and Health Sciences, Monash University, Melbourne, Victoria, Australia; 2 Department of Neurology, Alfred Health, Melbourne, Victoria, Australia; 3 Department of Neurology, Melbourne Health, Parkville, Victoria, Australia; 4 Department of Neurosurgery, Alfred Health, Melbourne, Victoria, Australia; 5 Department of Surgery, School of Translational Medicine, Melbourne, Victoria, Australia; Universite Paris-Saclay, FRANCE

## Abstract

Glioblastoma is the most common and aggressive form of primary brain cancer with a median survival of 15 months from diagnosis. The purinergic receptor P2X7 (P2X7R) is a regulator of several cell signalling pathways, and its expression is upregulated in glioblastoma. This study examined the expression and function of P2X7R in a human glioblastoma cell line, U251. We used a pharmacological antagonist of P2X7R, AZ10606120, to inhibit receptor function and delineate downstream consequences of receptor inhibition. Using RNA sequencing we demonstrated that P2X7R was expressed in the U251 cell line, harbouring both Y155H (Tyr to His) and E496A (Glu to Ala) single nucleotide polymorphism (SNP) mutations. The receptors functionality – namely its pore and channel conductance states were intact. Inhibition of P2X7R with small molecule antagonist AZ10606120, for 72 hours significantly decreased U251 cell number (p < 0.0001), and significantly increased tumour cell death, as evidenced by increased LDH release (p < 0.001). This reduction in tumour cell number was concentration-dependent, modelled by a least squares linear regression (R^2^ = 0.8221, IC50 = 17µM). The primary mode of cell death induced by AZ10606120 was shown to not be apoptosis, demonstrated through no significant changes in annexin V or cleaved caspase 3 staining in AZ10606120 treated cell versus control cells. Multiplex mRNA analysis demonstrated changes in genes associated with both apoptosis and pyroptosis, whilst a decrease in receptor-interacting serine/threonine-protein kinase 1 (RIPK1) expression along with an increase in TNFR1-associated death domain protein (TRADD) expression suggests potential involvement of the TRADD mediated RIPK1-independent necroptosis pathway. Collectively, this study describes several key characteristics of AZ10606120s acting as an anti-tumour small molecule pharmaceutical and highlights the potential of P2X7R inhibition as a novel therapeutic target in glioblastoma.

## Introduction

Glioblastoma is the most common and aggressive form of primary brain cancer accounting for 49.1% of all primary malignant brain tumours [1]. It has a prevalence of 3.23 per 100,000 people and is 1.58 times more common in men than women, with a median age at diagnosis of 65 [1–3]. The current standard therapy for glioblastoma is maximal safe surgical resection, followed by targeted cranial radiation and adjuvant chemotherapy, most commonly using temozolomide [4,5]. Since the introduction of temozolomide in 2005, there have been no major changes in the standard approach for glioblastoma treatment, and overall patient survival has remained poor [5–7]. Despite optimisations in clinical management and incorporation of molecular characteristics to inform diagnosis and patient prognostication, the 5-year survival rate is still only approximately 6.9% and median survival time is only 14–15 months from diagnosis [1,4,7,8]. Hence there is a pressing need for novel, safe and effective brain penetrant therapies to combat glioblastoma cell proliferation and increase patient survival.

One potential target is the purinergic receptor P2X7 (P2X7R). P2X7R is a multifunctional receptor which forms a transmembrane ion channel upon extracellular ATP binding, allowing for controlled influx of Na^+^ and Ca^2+^ ions with efflux of K^+ 9^. Continued stimulation with ATP results in formation of a large diameter pore that allows movement of proteins up to ~900 Da through the cell membrane, though the exact mechanism of this pore formation is still not understood [9–11]. P2X7R is expressed in a variety of cells including microglia, monocytes and macrophages [11,12] and has been implicated in a variety of cell functions including cellular growth, differentiation, cell death and neuroinflammatory processes [12–14]. Receptor expression is upregulated in several cancer settings, including glioblastoma, with studies demonstrating that higher expression confers a worse disease prognosis [15,16]. P2X7R function has been linked to multiple cellular processes, including modulation of energy metabolism [17,18], the release of IL-1β, NLRP3 assembly, activation of the PI3K/Akt inflammatory pathway, subsequent VEGF and activation of Erk1/2 signalling [12,19–21]. These pathways represent a variety of complex signalling cascades that can induce a variety of downstream trophic functions, such as angiogenesis, glycogen synthesis, cell survival, cellular proliferation, immune cell recruitment and cell migration [22–26]. Hence understanding of its function in glioblastoma is important both for development of novel treatments, as well as for its use as a potential biomarker of disease severity.

Whilst several P2X7R antagonists have been investigated in a range of disease contexts, a potential candidate for further study in cancer is AZ10606120 [27]. AZ10606120 is a small, high affinity antagonist with over 1,000-fold specificity for its target P2X7R. This specificity was demonstrated in a comparative binding study that measured the affinity of AZ10606120 binding to P2X7R compared to other partially homologous P2X receptor subtypes such as P2X4 and P2X6 [28]. Allosteric binding of AZ10606120 is thought to induce a conformational change in the 3D protein structure of the receptor, restricting the receptor function and inhibiting both the channel and pore conductance [28]. AZ10606120 has been investigated in several *in vivo* models of different cancer types, including mouse models of pancreatic cancer [29], mesothelioma [30], breast cancer [31], melanoma [16], and neuroblastoma [21]. It induced reductions in either tumour size or growth rate in each of these models, and its usage was associated with negligible side-effects in these models. In the context of glioblastoma, AZ10606120 has been shown to reduce cell numbers in both *in vitro* cultures of human high-grade gliomas, as well as in *in vitro* tumour stem cell cultures, further exhibiting its potential and need for comprehensive study [32–34].

The intracellular mechanisms that induce P2X7R mediated cell death are unknown. Understanding the mode of cell death mediated by AZ10606120 induced P2X7R inhibition and the mechanism underlying it is an integral step for its development as an anti-glioblastoma therapy. Multiple well-defined modes of cell death have been characterised in the literature [35]. The most well characterised regulated cell death pathways currently include apoptosis, pyroptosis and necroptosis. Apoptosis is the most common cell death pathway, thought of as the default regulated cell death pathway in most circumstances. It occurs through either intrinsic or extrinsic signalling pathways. The extrinsic pathway involves the sensing of danger signals through cell-surface death receptors such as TNFR1 or Fas, whilst the intrinsic pathway is activated by internal cell stressors which modulate proteins such as B-cell Lymphoma 2 (BCL-2) and BCL-2 like protein 4 (BAX) to induce apoptosis [36,37]. Both pathways converge to initiate the activation of the caspase 3/7 apoptotic molecular pathway resulting in controlled breakdown and disposal of the cell and its components [38,39]. Pyroptosis is associated with formation of the inflammasome construct in response to inflammasome sensor pattern recognition receptors such as absent in melanoma 2 (AIM2), and the release of proinflammatory cytokines such as interleukin-1 β (IL-1β) and interleukin-18 (IL-18) [40]. It’s been demonstrated that the PI3K-Akt pathway modulates pyroptosis, and therefore inhibition of P2X7R and subsequent modulation of this pathway could induce or upregulate pyroptosis mechanisms [41]. Necroptosis is preferentially induced in low energy cellular systems to ensure that cell death occurs when high levels of cell death signalling occur with limited energy resources [42–44]. This process is primarily initiated by Tumour Necrosis Factor (TNF) signalling though Tumour Necrosis Factor Receptor 1 (TNFR1) and subsequent recruitment of proteins such as TNFR1-Associated Death Domain (TRADD), Receptor-Interacting serine/threonine-Protein Kinase 1 (RIPK1) and TNF Receptor-Associated Factor 2 (TRAF2). Activation of this pathway has also been demonstrated through toll-like receptors, though with less mechanistic clarity [45,46]. Other modes of cell death that are less well characterised include processes such as autophagy, which involves the breakdown of cellular components via lysosomes, and necrosis, which is an ‘unregulated’ method of cell death in which cells swell and burst, releasing their contents into the surrounding environment. Elucidating the mode of cell death induced upon P2X7R inhibition would help decipher whether AZ10606120 or its derivatives could be effectively utilised, either alone or in synergy with currently available chemotherapies.

In this study we aimed to characterise the effect of AZ10606120 in the U251 human glioblastoma cell line. We found that P2X7R is expressed and identified that it harbours two SNP mutations, Y155H and E496A. The receptor was found to be functional in U251 cells, whilst its antagonism with AZ10606120, induced cell death in a dose-dependent manner. We identified changes in expression of several tumour promoting genes associated with P2X7R inhibition, as well as changes in key genes associated with apoptosis, pyroptosis and necroptosis. Collectively these results suggest limited involvement of apoptotic and pyroptotic pathways upon P2X7R inhibition, and provide evidence for potential involvement of necroptosis.

## Materials and methods

### U-251 MG cell culture and treatment

Human U-251 MG glioblastoma cells (U251; Sigma; Cat#09063001; RRID CVCL_0021) were maintained in Dulbecco’s modified Eagle’s Medium (DMEM; Lonza; Cat#BE12-604F), supplemented with 10% heat-inactivated FBS (ThermoFisher Scientific; Cat#10100147), 1% non-essential amino acids (NEAA; Sigma; Cat# M7145-100ML) and penicillin-streptomycin (50units/mL; ThermoFisher Scientific; Cat# 15070063). Cells were maintained at 37°C in a humidified incubator with 5% CO_2_ and routinely passaged at 80% confluency. Cells were grown to 70% confluency and then treated for 72 hours unless otherwise specified with either 50µM temozolomide (Sigma; Cat#T2577) or AZ10606120 made up in DMSO (Tocris Bioscience; Cat#3323) at specified concentrations. Equivalent DMSO amounts were tested against untreated cells with no significant changes observed in cytotoxicity, therefore untreated controls were utilised except unless otherwise specified.

### Immunocytochemistry

Cultured U251 cells were fixed using a 1:1 acetone/methanol solution at −20°C for 15 minutes. Cells were then washed and immersed in bovine serum albumin (4%; BSA; Sigma; Cat#A9418) for 1 hour at 37°C. Untreated fixed cells were stained with either rabbit anti-GFAP (Glial fibrillary acidic protein) (1:400; Dako; Cat#Z0334; RRID: AB_10013382) or rabbit anti-P2X7R FITC (Fluorescein isothiocyanate) conjugate (1:100; Sigma; Cat#P8997) primary antibody for 48 hours at 4°C. Cells treated with AZ10606120, and corresponding untreated control cells, were incubated with rabbit anti-cleaved caspase-3 (1:100; Cell signalling technology®; Cat#9664; RRID:AB_2070042) for 24 hours at 4°C. All primary antibody solutions were co-incubated with Triton-X 100 (0.1%; Sigma; Cat#T8787) to aid permeabilization. Stained samples were washed in PBS (Gibco; Cat#10010023) for 5 minutes in triplicate and then incubated with secondary antibodies, goat anti-rabbit Texas Red-X (1:200; ThermoFisher Scientific; Cat#T-2767; RRID:AB_2556776) for GFAP stained samples and goat anti-rabbit Alexa Fluor 488 (1:200; Invitrogen; Cat#A-11008; RRID:AB_143165) for cleaved caspase-3 samples, for 4 hours at 4°C. Samples were washed in triplicate as above and stained with 4′,6-diamidino-2-phenylindole nuclear stain (DAPI; 5µM; Sigma; Cat#MBD0015) for 1 hour at 25°C before being washed and mounted on slides with fluorescent mounting media (Dako; Cat# S3023). Samples were imaged using a Nikon A1r confocal microscope with a 40x magnification water immersion lens. All experiments were conducted with negative controls in the absence of primary antibodies.

### RNA extraction

Total RNA was extracted from culture U251 cells treated with AZ10606120 (15µM) and corresponding DMSO vehicle controls, using a QIAGEN RNeasy® Mini Kit (QIAGEN; Cat#74136) as per manufacturer’s protocol. Cells were gently scraped and centrifuged post treatment, before being lysed in manufacturer supplied buffer containing 1% β-Mercaptoethanol and homogenised using a TissueLyser LT (QIAGEN; Cat#85600). RNA was then extracted with an optional on-column DNase digestion step completed using the QIAGEN RNAse-Free DNase Set (QIAGEN; Cat#79254) as per manufacturer’s protocol. RNA concentration and purity were quantified spectrophotometrically (260/280 ratio) using a NanoDrop™ Lite spectrophotometer (ThermoFisher Scientific; Cat#ND-LITE-PR).

### RNA sequencing

RNA sequencing was carried out by Micromon Genomics (Monash University). 1 µg of total RNA extracted from control U251 culture samples underwent rRNA depletion and was then used to prepare RNA libraries using the NEBNext Ultra II Directional RNA Library Prep Kit for Illumina. The manufacturers protocol (v5.0_12/22) was followed with a target RNA fragment size of 200 bp, a 1/5 adapter dilution during the adapter ligation step and a total of 10 library amplification cycles. Resulting libraries were pooled at equimolar concentrations and prepared for sequencing using an DNBSEQ-G400 sequencer (MGI Tech) using a corresponding DNBSEQ-G400RS high-throughput sequencing kit (FCL PE100) (MGI Tech; Cat#1000019859) following manufacturers protocol. Resulting sequencing data was analysed by the Monash Bioinformatics Platform (Monash University). Quality control was performed using FastQC to ensure high-quality data. The resulting transcriptome was mapped to the NCBI human P2RX7 gene reference sequence (NM_002562.6; https://www.ncbi.nlm.nih.gov/gene/?term=NM_002562.6) via STAR RNA-seq software using 2-pass alignment to identify mutations.

### Live cell calcium imaging

Function of the P2X7R ion channel was assessed using the synthetic Ca^2+^ indicator Fluo-4 AM (ThermoFisher Scientific; Cat#F14201) and confocal microscopy. Cells were cultured in 4-well glass culture chambers until 70% confluency before being transferred to a Nikon 1Ar microscope with an incubator chamber maintained at 37°C and 5% CO_2_. Samples were incubated with Fluo-4 AM (1µM) for 30 minutes, with half the samples also preincubated with AZ10606120 (1µM) for 15 minutes prior to imaging to inhibit P2X7R function. Media was aspirated and replaced with DPBS with 1mM calcium (Gibco; Cat#14040133) and then imaging performed using a TimeSeries function to capture images over an 8-minute period (~1 image per second). 40 seconds after initialisation of TimeSeries imaging, 3’-O-(4-Benzoyl) benzoyl ATP (BzATP; 200µM; Sigma; Cat#B6396), a potent agonist of P2X7R, was added to stimulate the receptor. This concentration of BzATP was selected based on previous optimisation studies, and previous literature [21,47,30]. Channel function was subsequently quantified as the change in fluorescence intensity of individual cells before and after addition of BzATP (ΔF) and expressed as the relative change in fluorescence (ΔF/F_min_). This correlates to the change in Ca^2+^ concentration within the cell as an indicator of ion movement through the P2X7R ion channel. Each cell was identified by a region of interest (ROI) using FIJI (**F**IJI **I**s **J**ust **I**mageJ) image processing software (v1.56q). ~ 20–50 cells quantified per sample for 8 samples per treatment.

### YOPRO dye uptake imaging

Function of the P2X7R pore was assessed through measurement of cellular uptake of the nuclear dye YO-PRO-1 Iodide (YOPRO; ThermoFisher Scientific; Cat#Y3603). Cells were cultured in 8-well glass cell culture chambers until 70% confluency before being transferred to a live cell imaging stage on a Nikon 1Ar microscope, with cells maintained at 37°C and 5% CO_2_. Samples were incubated for 15 minutes with either YOPRO (1µM), YOPRO (1µM) & BzATP (200µM) simultaneously, or AZ10606120 (1µM) followed by subsequent simultaneous incubation with YOPRO (1µM) & BzATP (200µM) for an additional 15 minutes. After incubation the treated media was removed and replaced with prewarmed DPBS (Gibco; Cat#14287080) and samples were imaged immediately. Pore function was determined by quantifying the mean fluorescence intensity of each cell, identified by ROI using FIJI image analysis software. ~ 50 cells were quantified per sample for a total of 12 samples per treatment. Image analysis was performed blinded prior to statistical analysis.

### Lactate dehydrogenase cytotoxicity assay

Culture media supernatants from cells treated with AZ10606120 (50µM) or temozolomide (50µM) were assayed for cellular damage using a lactate dehydrogenase assay (LDH; Roche; Cat#11644793001). Samples were processed as per manufacturers protocol. Optical density was measured in duplicate per sample using a Multiskan Spectrum microplate reader (ThermoFisher Scientific) set to 450nm (with 540nm correction) with SkanIt software 2.4.2. Data was presented as the relative change in cytotoxicity of treated cells compared to untreated controls for 9 samples.

### Cell counts

Cultured cells in 24-well plates treated with temozolomide (50µM), AZ10606120 (5µM, 10µM, 15µM, 30µM or 50µM) or left untreated as controls, were fixed using a 1:1 acetone/methanol solution at −20°C for 15 minutes. Fixed cells were then stained with DAPI nuclear stain (5µM) for 1 hour at 25°C before being washed in triplicate with PBS. Cells were imaged on a Nikon TI-E fluorescence microscope at 20x magnification using an air objective lens. Cell counts were conducted using FIJI utilising the in-built “Analyse Particles” algorithm optimised to quantify the number of intact DAPI positive nuclei representative of the total number of viable cells. 15 randomly imaged fields from predetermined coordinates were quantified per sample for a total of 9 samples per treatment.

### Annexin V flow cytometry

Annexin V staining was quantified using flow cytometry to assess apoptosis in cultured cells treated with AZ10606120 (15µM) compared to untreated controls. This was performed using an Annexin V-PE Apoptosis detection kit (Invitrogen; Cat#88-8102-74) on cultured cells. To ensure a sufficient number of viable cells to be able to assess mode of cell death (apoptosis in this case), we treated cultured U251 cells with AZ10606120 (72 hours) plated in 6 well plates. Three wells from each plate were then combined for flow cytometry (number of cells = ~200,000 per experiment). Cultured treated cells were resuspended in annexin binding buffer and stained with the annexin V PE conjugated antibody following manufacturers protocols (Invitrogen) and maintained on at 4°C prior to analysis. Cells were counterstained with DAPI (5µM) acutely before analysis to identify dead cells. After excluding debris, cells that were DAPI and Annexin V double negative were deemed ‘live’. Those that were DAPI positive constituted ‘dead’ cells (necrotic), whilst those that were Annexin V positive constituted cells that have undergone apoptosis. Cell fluorescence was measured using a CytoFLEX Flow Cytometer (Beckman Coulter) with proportions of stained cells analysed using FlowJo analysis software v10.10.0.

### cDNA synthesis and quantitative PCR

Extracted total RNA samples from AZ10606120 treated and corresponding vehicle controls, underwent cDNA synthesis using a Transcriptor First Strand cDNA synthesis Kit (Roche; Cat#04897030001) as per manufacturer’s protocol. 1 µg of extracted RNA per sample was incubated with a combination of random hexamer primers and anchored-oligo(dT)18 primers to maximise reverse transcription. Reverse transcription reaction was carried out following cDNA protocol on a Verti 96-well thermocycler (Applied Biosystems). Quantitative PCR (qPCR) was completed using the Biomark HD system (Fluidigm) using a 48.48 Dynamic Array Integrated Fluidic Circuits (IFC) protocol. Genes analysed include several downstream of P2X7R activation, key genes associated with cell death pathways and 9 housekeeping genes (S1 Table). This was performed at Monash Health Translation precinct (MHTP) Medical Genomics Facility (Melbourne, Australia). Taqman assays were selected for each gene, provided as 20X forward and reverse primer and probe mixes at a concentration of 18µM and 4µM respectively. Quality checks were performed for all samples using SYBR GAPDH, followed by a 14-step preamplification step as detailed in Gene Expression Preamp with Fluidigm Preamp MasterMix and Taqman Assays (Quick Reference PN 100–5876 B1). Assays and samples were loaded onto a 48.48 gene expression IFC using 5 µL of each assay at 10X and 5 µL of sample diluted 1:5 with TE buffer. Data was analysed using Fluidigm Real-Time PCR analysis software (V4.1.1). Cycle threshold (Ct) values for genes of interest were normalised to a geometric mean of housekeeping genes (S1 Table) [48]. Ct values were converted into fold change in gene expression relative to the control group using the 2^-ΔΔCt^ method [49]. Fold change (FC) values were subsequently log transformed (Log2; Log fold change; LFC) to normalise data for statistical analysis, with the change in expression of treatment relative to control for each gene graphically represented.

### Statistical analysis

All statistical tests were conducted using the GraphPad Prism 9 software. Normality of data sets was assessed using D’Agostino & Pearson normality tests. Outliers were removed where applicable using the ROUT method of outlier removal. Non-parametric data was analysed via a Mann Whitney U test or a Kruskal-Wallis test with post hoc Dunn’s multiple comparisons dependant on the number of treatment groups. Parametric data was analysed via either unpaired t-tests or one-way ANOVA with post hoc Tukey’s HSD tests, dependant on the number of treatment groups. Additionally, a non-linear least squares regression was used to model a dose-response relationship. Data was expressed as mean ± SEM with statistical significance set at p < 0.05.

## Results

### Human U251 glioblastoma cells express P2X7R with both Y155H and E496A functional mutations

**Fig 1:**
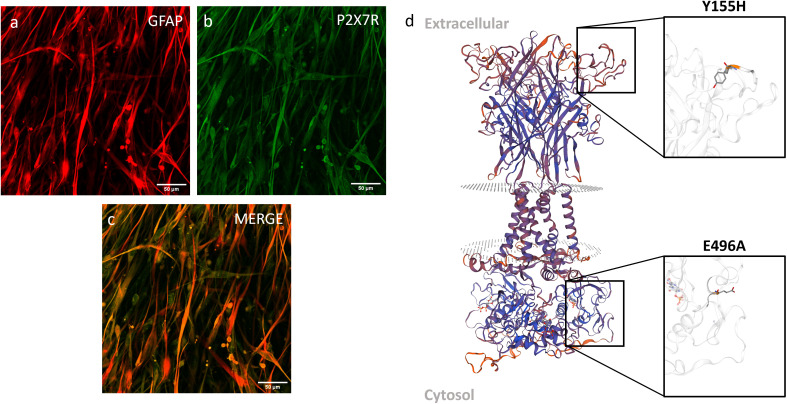
P2X7R expression and sequence mutations demonstrated in the U251 glioblastoma cell line. U251 cell cultures showing **(a)** primary rabbit anti-GFAP and secondary goat anti-rabbit Texas Red and **(b)** primary anti-P2X7R FITC conjugate. **(c)** Merged image demonstrating co-expression of GFAP and P2X7R stains homogenously expressed in U251 cells. Images taken at 40x magnification. Scale bars = 50µm. **(d)** Representative protein structure of human P2X7R (P2RX7: Chromosome 12: 121132876 - 121188032 (+)) with locations of functional single nucleotide polymorphisms (SNPs) (Y155H; TAT - > CAT and E496A; GAG - > GCG) present in P2X7R expressed in the U251 glioblastoma cell line as determined by Next Generation Sequencing (NGS). Protein structure modelled via SWISS-MODEL Repository [50].

Expression of P2X7R in the U251 cell line was identified via immunocytochemistry and RNA sequencing. Using RNA sequencing we also identified two SNP mutations that would affect receptor function (Fig 1). Immunocytochemistry demonstrated expression of both GFAP (used as a marker of differentiated glioma cells) (Fig 1a) and P2X7R (Fig 1b) with co-expression of these markers demonstrated throughout the cell (Fig 1c). RNA sequencing was performed on total RNA extracted from U251 cell cultures and the P2X7R gene (P2RX7) assessed for mutations in comparison to the NCBI human P2RX7 gene reference sequence (NM_002562.6; https://www.ncbi.nlm.nih.gov/‌gene/?term=NM_002562.6). In comparison to the reference sequence, the U251 P2X7R transcriptome expressed both Y155H (TAT - > CAT) and E496A (GAG - > GCG) SNPs (Fig 1d).

### The P2X7R ion channel and pore functions are intact in the U251 cell line

**Fig 2:**
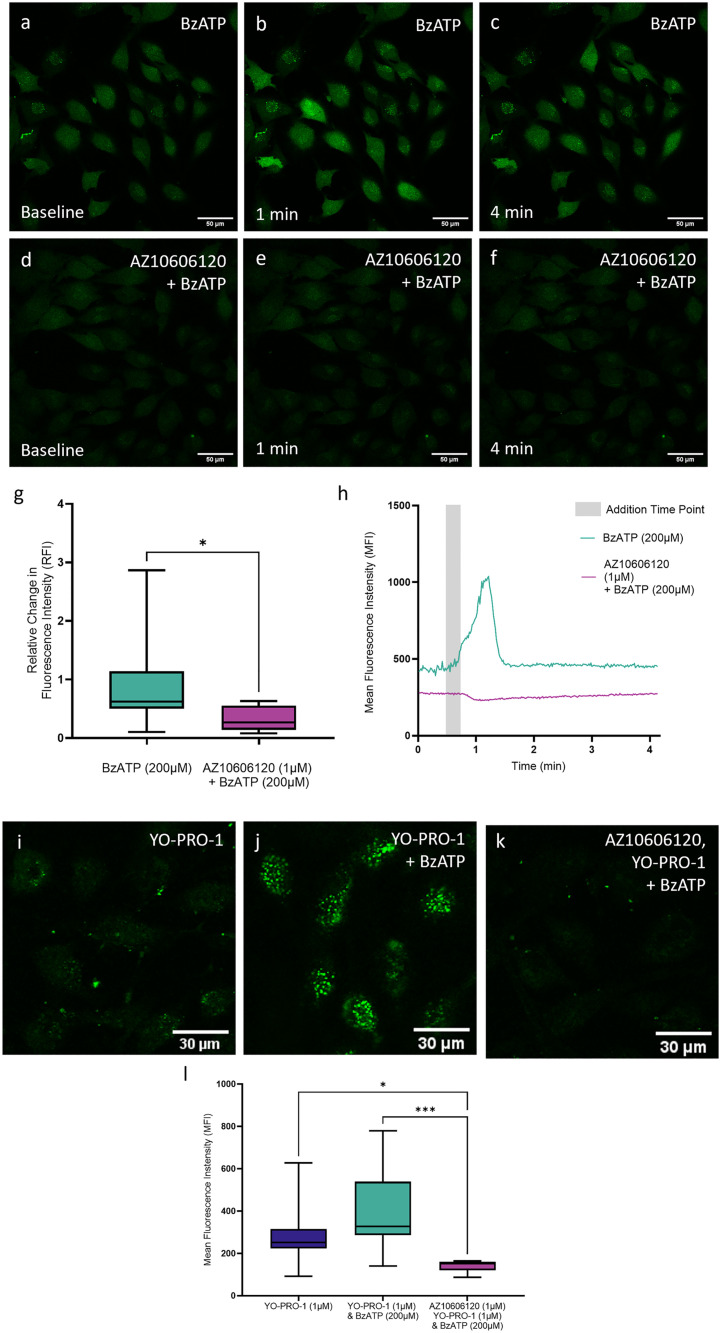
P2X7R ion channel and pore are functional in the U251 glioblastoma cell line. Representative time series images of U251 glioblastoma cells stained with fluorescent synthetic Ca^2+^ indicator Fluo-4 AM. **(a-c)** Cells stimulated with BzATP (200μM) or **(d-f)** pretreated with AZ10606120 (1μM) and subsequently stimulated with BzATP (200μM). Images taken at 40x magnification. Scale bars = 50µm **(g)** P2X7R ion channel function demonstrated in U251 human glioblastoma cells stained with the fluorescent synthetic Ca2 + indicator dye Fluo-4 AM. Activity of the ion channel was quantified via measuring the change of fluorescence intensity upon addition of P2X7R agonist BzATP (200µM), relative to fluorescence prior to addition (baseline). An increased change in fluorescence intensity was shown in cells stimulated with BzATP (200µM) compared to cells pretreated with P2X7R antagonist AZ10606120 (1µM; 15 mins) prior to stimulation with BzATP, which showed significantly diminished fluorescence changes upon BzATP (200μM) addition. Data expressed as median and IQR. 20-50 cells were analysed per sample with a total of n = 8 samples per group. * p < 0.05; Mann Whitney U Test. **(h)** Representative line graph of the change in fluorescence intensity over time of a cell stimulated with BzATP (200μM) and a cell pretreated with AZ10606120 (1μM) prior to BzATP addition, highlighting that agonist stimulation results in a specific calcium increase that is not seen in the presence of the antagonist. **(i-l)** P2X7R pore channel function demonstrated in U251 human glioblastoma cells stained with the fluorescent nuclear dye YOPRO. Activity of the P2X7R pore was quantified via measuring uptake of YOPRO in unstimulated cells compared to cells stimulated with P2X7R agonist BzATP (200µM), and cells pretreated with P2X7R antagonist AZ10606120 (1µM; 15 mins) prior to stimulation with BzATP. YOPRO fluorescence was evident both at baseline as well as in cells stimulated with BzATP (200µM) and was significantly reduced in cells pretreated with AZ10606120 (1µM) before BzATP stimulation. Data expressed as median and IQR. Approximately 50 cells were analysed per sample with a total of n = 12 samples per group. * p < 0.05; *** p < 0.001; Kruskal Wallis Test. **(i-l)** Representative images of U251 cells treated with **(i)** YOPRO (1µM) only, **(j)** YOPRO (1µM) & BzATP (200µM) simultaneously and **(k)** acutely pretreated with P2X7R antagonist AZ10606120 (1µM; 15 minutes) followed by YOPRO (1µM) & BzATP (200µM) addition. Images taken at 60x magnification. Scale bars = 30µm.

To determine functionality of P2X7R, activity of both the ion channel and pore functions of P2X7R were quantified in U251 cells through live cell calcium imaging and dye uptake imaging respectively. To evaluate ion channel function transient calcium signals were visualised through Fluo-4 AM fluorescence and quantified over time in cells stimulated with P2X7R agonist BzATP (Fig 2a-2c) and those pretreated with AZ10606120 prior to BzATP stimulation (Fig 2d-2f). BzATP stimulated cells demonstrated transient calcium waves indicative of ion channel activity, whilst pretreatment with AZ10606120 significantly diminished these responses (Fig 2g and 2h) (*p < 0.01). Pore function was quantified through uptake of the nuclear dye YOPRO. YOPRO uptake was evident in unstimulated cells (Fig 2i) indicating a combination of both cellular uptake mechanisms such as endocytosis, and potential P2X7R functionality in unstimulated cells. This uptake was increased upon stimulation with BzATP, highlighting P2X7R functionality (Fig 2j) and significantly decreased compared to both stimulated and unstimulated cells, in cells pretreated with AZ10606120 prior to BzATP stimulation (Fig 2k and 2l)(*p < 0.05; ***p < 0.001). These results indicate that both the P2X7R ion channel and pore are functional in the U251 cell line, demonstrated by stimulation with the P2X7R agonist BzATP, and these functions can be inhibited by treatment with P2X7R antagonist AZ10606120.

### Inhibition of P2X7R with AZ10606120 reduces U251 cell number and increases extracellular LDH levels

**Fig 3:**
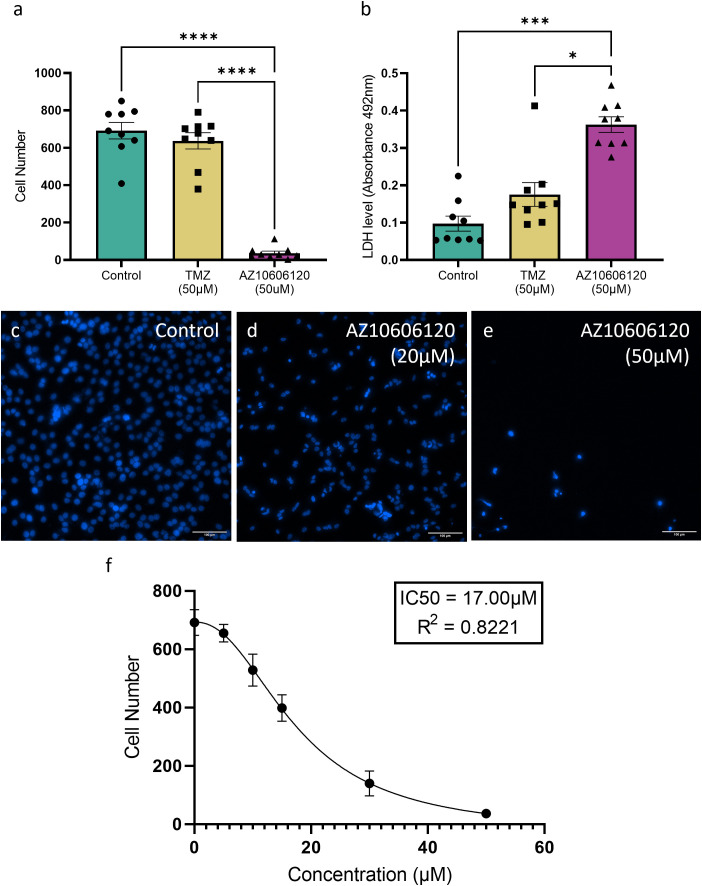
AZ10606120 treatment significantly increased cytotoxicity and reduced cell number in a dose-dependent manner. **(a)** Cell numbers quantified by manual cell counts of viable U251 cells post-treatments. Cell numbers were significantly decreased in cells after treatment with AZ10606120 (50µM) compared to both temozolomide (TMZ; 50µM) treatment and untreated controls. Data is represented as mean ± SEM, n = 9 samples per treatment. ****p < 0.0001; Kruskal Wallis test. **(b)** Cytotoxicity quantified via LDH assay of U251 culture supernatants post-treatment. LDH levels were significantly increased in culture supernatants treated with AZ10606120 (50µM) compared to temozolomide (50µM) treatment and untreated controls. Data is represented as mean ± SEM, n = 9 samples per treatment. *p < 0.05, ***p < 0.001; One-way ANOVA. **(c-e)** Representative images of DAPI (5µM) stained U251cultures **(c)** untreated controls, **(d)** treated with AZ10606120 (20µM) and **(e)** treated with AZ10606120 (50μM). Images taken at 20x magnification. Scale bars = 100µm **(f)** Dose-response relationship between AZ10606120 treatments and cell number in U251 cell cultures demonstrating that increasing AZ10606120 concentrations (0-50µM) correlated to decreasing cell number. This relationship was modelled using a non-linear least squares regression model for a sigmoidal curve. Data expressed as mean ± SEM for n = 9 samples per dose. IC50 = 17µM; R 2 = 0.8221.

Cell viability was determined by comparing untreated U251 cell cultures, to cultures treated with either AZ10606120 (50µM), or the conventional chemotherapy temozolomide (50µM) for 72 hours with cell numbers subsequently quantified. LDH levels were also assessed in the culture supernatants of these samples harvested post-treatment period, as a measure of cytotoxicity. AZ10606120 treatment significantly decreased cell number whilst also significantly increasing LDH levels in comparison to both untreated cultures and temozolomide treatment. Temozolomide also showed no significant difference in either cell number or LDH release compared to untreated controls (Fig 3a and 3b) (*p < 0.05; ***p < 0.001; ****p < 0.0001). Following this, a range of AZ10606120 treatment concentrations were investigated to determine the relationship between treatment and cell number (Fig 3c-3e). A clear dose-response relationship was established between AZ10606120 treatment and culture cell number, with increasing AZ10606120 concentration resulting in decreased cell number. A non-linear least squares regression model was used to model the relationship which was effectively described using a sigmoid curve with an R 2 = 0.8221 and an IC_50_ of 17.00µM (Fig 3f).

### AZ10606120 treatment does not induce apoptosis

**Fig 4:**
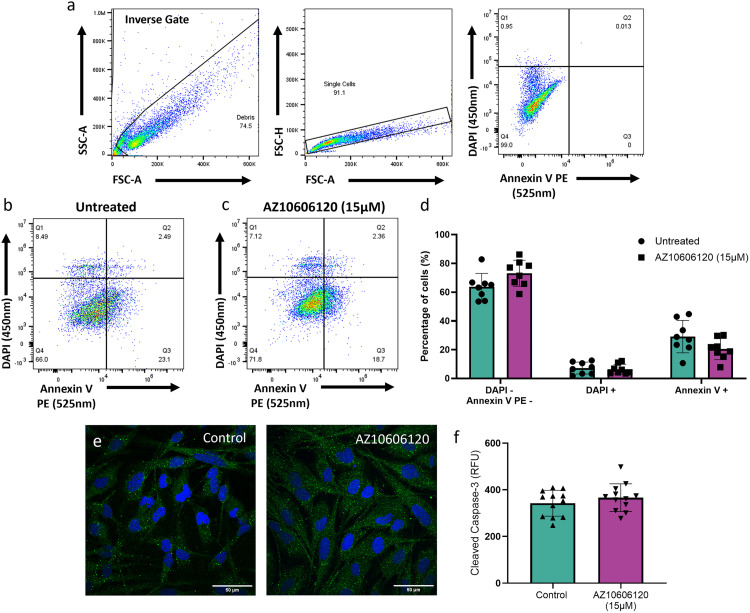
AZ10606120 treatment does not significantly increase apoptosis mediated cell death. Apoptosis was investigated in AZ10606120 treated U251 cells via annexin V staining quantified via flow cytometry. **(a)** Flow cytometry gating strategy in which debris was excluded, single cells were selected for, and all remaining cells, including dead/dying cells, gated as the negative population. **(b,c)** Representative dot plots showing fluorescence signals and gating of **(b)** untreated and **(c)** AZ10606120 (15µM; 72 hours) treated U251 cells stained with DAPI (450/50nm filter) and Annexin V PE (525/30nm filter). **(d)** Apoptosis was measured by determining the percentage of double negative cells (Annexin V PE-, DAPI-), necrotic (dead) cells (DAPI+) and apoptotic cells (Annexin V PE+). Quantified cell proportions were not significantly different in samples treated with AZ10606120 (15µM; 72 hours) compared to untreated controls. Data is represented as mean ± SEM, n = 8 samples per treatment. Unpaired t-tests. **(e)** Apoptosis was subsequently investigated via cleaved caspase 3 staining. Representative images of U251 cultures stained with primary rabbit anti-cleaved caspase-3, secondary anti-rabbit Alexa flour 488 antibody and subsequent DAPI (5µM) for untreated control cultures (first panel) and cultures treated with AZ10606120 (15µM; 72 hours; second panel). Images taken at 60x magnification. Scale bars = 50µM for all images. **(f)** Cleaved caspase-3 staining was quantified by measuring the total green fluorescence (RFU) intensity in each image. There was no significant difference in cleaved caspase-3 expression between AZ10606120 (15µM) treated cells and untreated controls. Data expressed as mean ± SEM. Approximately 20 cells were analysed per sample for n = 12 images per treatment. Unpaired t-test.

The mechanism underpinning the cytotoxicity demonstrated by AZ10606120 treatment is currently undefined. Historically, P2X7R function has been associated with apoptosis in different contexts [51,52]. To evaluate apoptosis in U251 cells, the apoptotic markers annexin V and cleaved caspase-3 were quantified. Annexin V is a protein that binds the phosphatidylserine residues exposed on the cell surface during the early stages of apoptosis and its levels as measured by flow cytometry are used as a marker of apoptosis [53]. Cells treated with AZ10606120 showed no changes in the proportion of annexin V positive cells relative to those in control samples (Fig 4a-4d). The intracellular expression of the protease caspase 3 was used as an alternative marker of apoptosis. Caspase-3 catalyses cleavage of key proteins of the apoptotic pathway, and presence of its active form cleaved caspase-3 is a reliable marker of apoptosis [39,54]. Quantification of relative fluorescence (RFU) of AZ10606120 treated cells demonstrated no significant differences in cleaved caspase-3 expression relative to control cells (Fig 4e and 4f). The minimal changes in both annexin V and cleaved caspase levels upon AZ10606120 treatment, demonstrate that AZ10606120 induced cytotoxicity is unlikely apoptotic in nature.

### Assessing the mechanisms underlying AZ10606120 induced cytotoxicity – A potential role for RIPK1-independent TRADD mediated necroptosis

**Fig 5:**
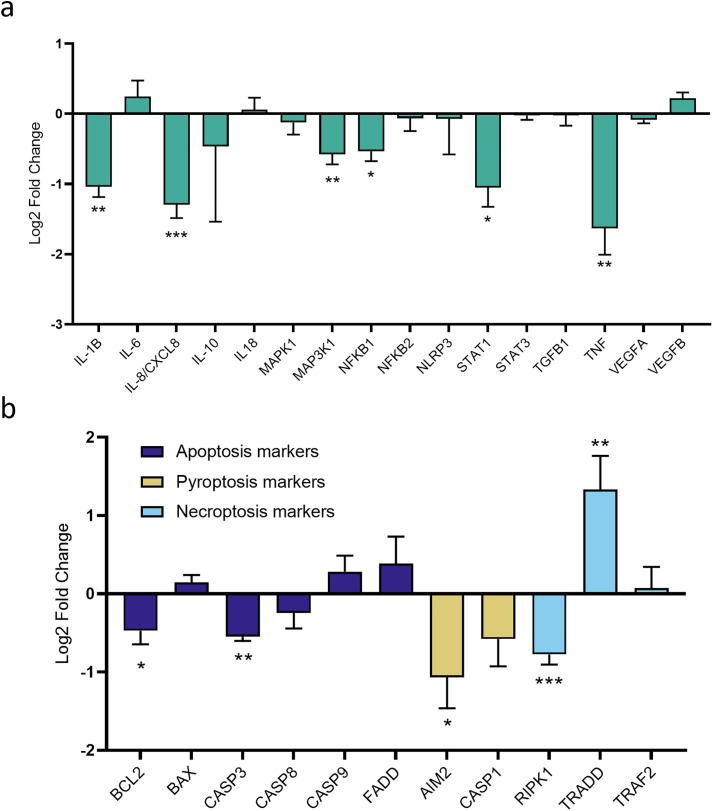
Investigations of cell death mechanisms induced by AZ10606120 treatment indicate involvement of RIPK1-independent TRADD mediated necroptosis. **(a)** Gene expression of key genes associated with known downstream pathways of P2X7R activation in U251 cultures treated with AZ10606120 (15µM) relative to vehicle controls. AZ10606120 treatment induced downregulation of several key genes, demonstrating inhibition of P2X7R inhibits several well characterised inflammatory pathways (IL-1B, IL-8, MAP3K1, NFKB1, STAT1, TNF). Fold change was calculated using 2-ΔΔCt method relative to control samples. Data was transformed to log2 fold change (LFC) and expressed as LFC ± SEM, n = 12 samples per treatment. *p < 0.05; **p < 0.01; ***p < 0.001. Unpaired t-tests & Mann-Whitney U tests. **(b)** Gene expression of several genes integral to the programmed cell death pathways of apoptosis, pyroptosis and necroptosis were investigated in U251 cultures treated with AZ10606120 (15µM) relative to vehicle controls. Gene expression data was quantified via high-throughput qPCR analysis. Fold change was calculated using the 2-ΔΔCt method relative to vehicle control samples. Genes associated with apoptosis and pyroptosis were demonstrated to be downregulated (BCL2, CASP3, AIM2) due to AZ10606120 treatment, whilst genes associated with necroptosis indicated potential upregulation of RIPK1-independent TRADD mediated necroptosis mechanisms (RIPK1, TRADD) in AZ10606120 treated samples. Data was transformed to log2 fold change (LFC) and expressed as LFC ± SEM, 12 samples per treatment. *p < 0.05; **p < 0.01; ***p < 0.001. Unpaired t-tests & Mann-Whitney U tests.

The mechanisms underlying AZ10606120 induced cytotoxicity were further investigated via characterisation of mRNA expression. A range of genes associated with the pathways downstream of P2X7R activation, as well as genes associated with the programmed cell death pathways of apoptosis, pyroptosis and necroptosis, were quantified through high-throughput qPCR. AZ10606120 treatment downregulated gene expression of several key genes associated with pathways downstream of P2X7R activation, including IL-1β (fold decrease = 0.736; LFC = −1.036), Interlukin-8 (IL-8/CXCL8; fold decrease = 0.408; LFC = −1.843), Mitogen-Activated Protein Kinase Kinase Kinase 1 (MAP3K1; fold decrease = 0.708; LFC = −0.576), Nuclear Factor Kappa-light-chain-enhancer of activated B cells 1 (NF-κB1; fold decrease = 0.723; LFC = −0.535), Signal Transducer and Activator of Transcription 1 (STAT1; fold decrease = 0.578; LFC = −1.050), and TNF (fold decrease = 0.422; LFC = −1.631). (Fig 5a) (*p < 0.05; *p < 0.01; ***p < 0.001). Gene expression analysis of genes associated with programmed cell death pathways demonstrated that AZ10606120 treatment significantly downregulated the expression of BCL2 (fold decrease = 0.774; LFC = −0.472), Caspase-3 (CASP3; fold decrease = 0.689; LFC = −0.549), AIM2 (fold decrease = 0.631; LFC = −1.066), and RIPK1 (fold decrease = 0.612; LFC = −0.773), whilst upregulating expression of TRADD (fold increase = 4.834; LFC = 1.333) (Fig 5b) (*p < 0.05; *p < 0.01; ***p < 0.001).

Though varied, collectively these results indicate that AZ10606120 induced U251 cellular cytotoxicity is less likely to be predominately mediated via apoptosis or pyroptosis as a primary mechanism, and implicates a potential upregulated role of RIPK1-indepednant TRADD mediated necroptosis. They also highlight that P2X7R inhibition results in significant downregulation in gene expression of several well characterised components of both inflammatory and tumour promoting pathways.

## Discussion

The results of this study characterised several elements of P2X7R expression and function in U251 glioblastoma cells. Firstly, we validated the expression of P2X7R in the U251 cell line and determined that P2X7R harbour 2 specific SNPs, the Y155H and E496A mutations. We demonstrated that the receptors ion channel and pore conductance states were both intact. Inhibition of P2X7R with the small molecule antagonist AZ10606120, resulted in a decreased U251 cell number and increased LDH release indicating cell death. Importantly, AZ10606120 was more efficacious in reducing U251 cell number compared to the conventional chemotherapy temozolomide. Quantification of both annexin V and cleaved caspase-3 levels indicated that the mode of cell death induced by AZ10606120 was unlikely to be apoptosis. Changes in gene expression upon AZ10606120 treatment were varied, with downregulation of both pro- and anti-apoptotic genes observed. Changes in genes associated with necroptosis provided evidence for a RIPK1 independent TRADD mediated necroptosis pathway as a potential mechanism of AZ10606120 induced cell death. Future studies should focus on investigating necroptosis, in addition to cellular metabolism mechanisms including, energy biogenesis, mitoptosis induced autophagy, and mitochondrial permeability transition (MPT)-driven necrosis [55,56].

P2X7R expression demonstrated in our studies is consistent with several previous studies that demonstrated P2X7R expression in a range of glioblastoma models, including the human U251, U87, U138 cell lines, the rat C6 cell line and human glioma cultures [57–59]. The Y155H mutation expressed by P2X7R in the U251 cell line has been associated with decreased cell surface expression of P2X7R as well as reduced ATP evoked calcium channel currents, demonstrated in both WT and P2X7R transfected HEK293 cells [60]. In contrast, the effects of the E496A mutation are unclear, with one study demonstrating that the E496A mutation resulted in no changes in receptor activity and function [61], whilst a separate study utilising the same experimental conditions indicated functional impairment [62]. Contrary to this, our data revealed that P2X7R is expressed and functional (both ion channel and pore conductance states) in the U251 cell line despite having the same SNP. Previous studies in the U251 cell lines and similarly demonstrated both ion channel functionality [57] and pore formation [57] of P2X7R. Considering the mutations demonstrated in P2X7R in this context, these results suggest that expression of these mutations does not completely inhibit P2X7R functions, though future patch clamp studies are needed to elucidate whether the receptor’s ion channel or pore functions are diminished or enhanced compared to wild-type P2X7R.

P2X7R has been demonstrated as both intact and non-functional in cancer cells. Studies conducted in multiple cancer cell lines (RPMI-8226, SK-MEL-5, PC3, DU145, LNCaP, Kelly and Ramos) hypothesised that a truncated non pore forming P2X7R variant (non-functional P2X7R; nfP2X7R) is overexpressed in the cancer setting and is therefore responsible for the trophic effects observed in these cells [63,64]. Under this hypothesis, the intact functional P2X7R receptor is often indicated to be cytolytic rather than trophic [65,66]. Furthermore, functional splice variant isoforms of P2X7R, known as P2X7RA and P2X7RB have also been described, and in glioblastoma specifically, shown to be differentially expressed and associated with distinct functions [66]. These studies highlight the varied roles that P2X7R can have in both physiological and disease contexts, and the importance of characterising the specific P2X7R variants and mutations present, as well as overall expression and function of the receptor. Our results demonstrate that in the glioblastoma U251 cell line two specific SNPs were present in the P2X7R gene, with P2X7R demonstrated as both expressed and functional. This highlighted that functional P2X7R is pro-tumour in the U251 cell line, and that its inhibition as demonstrated, results in a reduction in tumour cell number.

Downstream effects of P2X7R antagonism were investigated through prolonged (72 hour) treatment with AZ10606120, which demonstrated a significant decrease in cell number and a significant increase in LDH release in U251 cells. These results align with several studies that demonstrate the anti-tumour effect of AZ10606120 treatment. This includes our previous published work in human glioblastoma primary cultures [32,34] and stem cell cultures [33], as well as studies of melanoma [16], pancreatic cancer [29], mesothelioma [30], breast cancer [31] and neuroblastoma [21]. Both the increase in LDH release and decrease in cell number were significantly enhanced with treatment of AZ10606120 compared to temozolomide treatment. A dose-response relationship for AZ10606120s effect was modelled for its effect on cell number, with an IC50 of 17µM determined for this cell line. These results highlight the efficacy of AZ10606120 and its potential as a therapy in glioblastoma.

We next investigated the mode of cell death induced by AZ10606120 in the U251 cell line. Previous studies have shown P2X7R function to be linked with apoptosis in different cell lines [67–69], therefore apoptosis was initially investigated. Quantification of both annexin V and cleaved caspase-3, key markers of apoptotic activity, demonstrated no significant differences upon AZ10606120 treatment. Whilst previous data on the method of AZ10606120 induced cell death is limited, these results do align with one study that indicated AZ10606120 treatment did not influence the caspase 3/7 apoptotic pathway in pancreatic ductal adenocarcinoma (PDAC) cells [69].

A panel of genes was subsequently examined using qPCR, to quantify their relative gene expression changes upon AZ10606120 treatment. Expression of several genes associated with pathways downstream of P2X7R activation were significantly downregulated by AZ10606120 treatment. This includes IL-1β, IL-8, MAP3K1, NF-κB1, STAT1 and TNF. These genes are associated with various pro-tumour processes including proliferation, migration, angiogenesis, and cell survival. Specifically, IL-1β, NF-κB1 and TNF have all been associated with promoting an inflammatory microenvironment in cancer settings leading to proliferation, angiogenesis, and cell survival [6,70,71], whilst also being linked with specific cell death mechanisms as described above. IL-8 is linked to tumour progression and invasion [72], whilst MAP3K is part of a signalling pathway linked to proliferation, invasion and angiogenesis [73]. Finally, STAT1 has been described as both a tumour suppressor and an oncogene in different contexts [74]. Downregulation of all these genes due to AZ10606120 treatment is indicative of a reduction in the associated pro-tumour pathways that are linked to P2X7R activation. This result highlights the potential effectiveness of inhibiting P2X7R in the cancer setting, as it can alter numerous well described tumour promoting pathways downstream of the receptor.

Expression of genes associated with programmed cell death pathways were also significantly modulated by AZ10606120 treatment. Both the pro-apoptotic CASP3 gene and the anti-apoptotic BCL2 gene demonstrated significant downregulation upon AZ10606120 treatment. Considering no changes were seen in annexin V and cleaved caspase-3 at a protein level, whilst the anti-apoptotic BCL2 was downregulated, collectively these results still indicate that apoptosis is unlikely to be a key mode of cell death induced by AZ10606120 inhibition of P2X7R. The downregulation of BCL2, could be a compensatory response to suppression of CASP3 activity, however further investigations into these pathways is needed to investigate these appropriately. AIM2, a key initiator of the inflammasome complex that defines pyroptosis [40] was also significantly downregulated. This in conjunction with the downregulation of IL-1β and NF-κB1 previously discussed, indicates that pyroptosis is also not significantly induced by AZ10606120 treatment. Genes associated with necroptosis also showed significant expression changes. RIPK1 expression was downregulated, whilst TRADD expression was significantly upregulated. Downregulation of RIPK1 indicates it is unlikely that AZ10606120 treatment induces RIPK1-dependant necroptotic cell death [45]. The observed changes in both RIPK1 and TRADD expression upon AZ10606120 treatment are consistent with an alternate necroptosis pathway induced by TNF, termed TRADD mediated RIPK1-independent necroptosis [75]. However, considering TNF is also significantly downregulated, it is unclear whether the upregulation of TRADD is indicative of an increase of this necroptotic pathway. These changes could alternatively be activated by paracrine TNF release, or be a compensatory mechanism for the downregulation of TNF caused by P2X7R inhibition. This is not determinable in the single cell culture setting and therefore should be investigated in future comprehensive studies.

These results are limited to gene expression changes only, and do not rule out the potential involvement of other cell death mechanisms such as mitoptosis, autophagy or necrosis. Several studies highlight the trophic role of P2X7R in cancer contexts, specifically inducing proliferation of immune and cancer cells, promoting an inflammatory microenvironment which facilitates cell survival, suppressing immune surveillance, and inducing changes in metabolic pathways that favour tumorigenesis [65,76,77]. Therefore, a hypothesised mechanism for the cell death observed upon P2X7R antagonism is inhibition of these trophic functions., This leads to a reduced ability for cells to survive and proliferate resulting in the observed cell death. Alternatively, as P2X7R is also associated with metabolic pathways, including changes to energy metabolism [78] and mitochondrial dysfunction [79]. Therefore, another possible mechanism underpinning the cell death observed is that inhibition of P2X7R leads to disruption of mitochondrial potential, resulting in mitoptosis and subsequent cellular degradation, inducing autophagic cell death [55,80,81]. This could be in part due to disruption of ATP release, as P2X7R has been demonstrated to control release of intracellular ATP through the P2X7R pore as a method of autocrine function [77,82]. Alternatively, receptor inhibition could impact cell cycle regulation leading to cellular breakdown and subsequent necrosis [81]. P2X7R activation has been shown to accelerate cell cycle entry, increasing the proportion of cells in S-phase, potentially by influencing transient calcium signals necessary for G1/S phase progression [83]. In general, alterations of energy biogenesis within the cell, disruptions of mitochondria and arrest of cell cycling pathways could all lead to cell death and therefore these alternative modes of cell death, are clear targets for future research. Future studies should investigate these mechanisms along with further validation of apoptosis, pyroptosis and necroptosis, to elucidate the exact mechanisms underpinning how inhibition of P2X7R can lead to cell death.

This study is limited due to it being conducted solely in the U251 cell line. Although a single cell line provides a homogenous population of cells where direct effects of pharmacological agents can be studied, it’s not reflective of the intricate *in vivo* environment, and is limited in its ability to be generalised to glioblastoma overall. Further to this, only 1 time point (72 hours post treatment) was investigated in the cell death studies. This incubation time was chosen to ensure sufficient time for downstream intracellular processes to be observed upon receptor function. Future studies should aim to provide a more detailed temporal profile of the molecular and functional changes induced by AZ10606120. Furthermore, future research should aim to replicate and broaden this work in multiple human glioblastoma cell lines, animal models and human glioblastoma cultures to both ensure results are generalisable to the overall pathology of glioblastoma, and validate the demonstrated outcomes are relevant to the clinical setting.

## Conclusion

This study assessed several key aspects of P2X7R inhibition by AZ10606120 in the human U251 glioblastoma cell line. It demonstrated that P2X7R is expressed and harbours 2 distinct SNPs, and that despite this the receptor channel and pore functions are intact. It also determined a clear dose-response relationship between AZ10606120 treatment and cell death. We showed that inhibition of the receptor resulted in downregulation of various key markers of cell trophism and survival, highlighting the pro-tumour roles of the receptor. Our data revealed that the mode of AZ10606120 mediated tumour cell death is unlikely that of apoptosis nor pyroptosis. TRADD mediated RIPK1-independent necroptosis could be a possible pathway by which AZ10606120 mediated cell death is induced, though this needs further clarification. This along with energy metabolism dysregulation and cell cycle inhibition are of particular interest for future research. In totality, our study provides several exciting initial insights in understanding the role of P2X7R in glioblastoma and paves the way for future investigations of AZ10606120 as a potential therapeutic strategy to combat glioblastoma.

## Supporting information

S1 File Supporting datasets for all figures. (ZIP)

S1 Table List of Taqman Assay IDs for genes quantified via high-throughput qPCR. (TIF)

## Abbreviations

AIM2: Absent in melanoma 2
ANOVA: Analysis of Variance
ATP: Adenosine Triphosphate
BCL2: B-cell lymphoma 2
BzATP: 3’-O-(4-Benzoyl)benzoyl ATP)
CASP3: Caspase-3
Ct: Cycle Threshold
DAPI: 4′,6-diamidino-2-phenylindole
DMEM: Dulbecco’s Modified Eagles Medium
DMSO: Dimethyl Sulfoxide
FC: Fold change
FIJI: IJI Is Just ImageJ
FITC: Fluorescein isothiocyanate
GAPDH: Glyceraldehyde 3-phosphate dehydrogenase
GFAP: Glial Fibrillary Acidic Protein
IL-1β: Interleukin 1 beta
IL-8/CXCL8: Interleukin 8/ Chemokine ligand 8
LDH: Lactate Dehydrogenase
LFC: Log2 fold change
MAP3K1: Mitogen-activated protein kinase kinase kinase 1
NF-κB: Nuclear factor kappa-light-chain-enhancer of activated B cells
NF-κB1: NF-kappa B Nuclear Factor subunit 1
P2X7R: Purinergic P2X Receptor 7
RIPK1: Receptor-interacting serine/threonine-protein kinase 1
ROI: Region of interest
STAT1: Signal Transducer and Activator of Transcription 1
TMZ: Temozolomide
TNF: Tumour Necrosis Factor
TRADD: Tumour necrosis factor receptor type 1-associated DEATH domain protein
YOPRO: YO-PRO-1 Iodide
