# Supplementary figures and images for: Purinergic P2X receptor 7 (P2X7R) inhibition induced cytotoxicity in glioblastoma
Source: PLoS One. 2025 Sep 16;20(9):e0332212. doi: 10.1371/journal.pone.0332212 (PMC12440200; doi:10.1371/journal.pone.0332212)

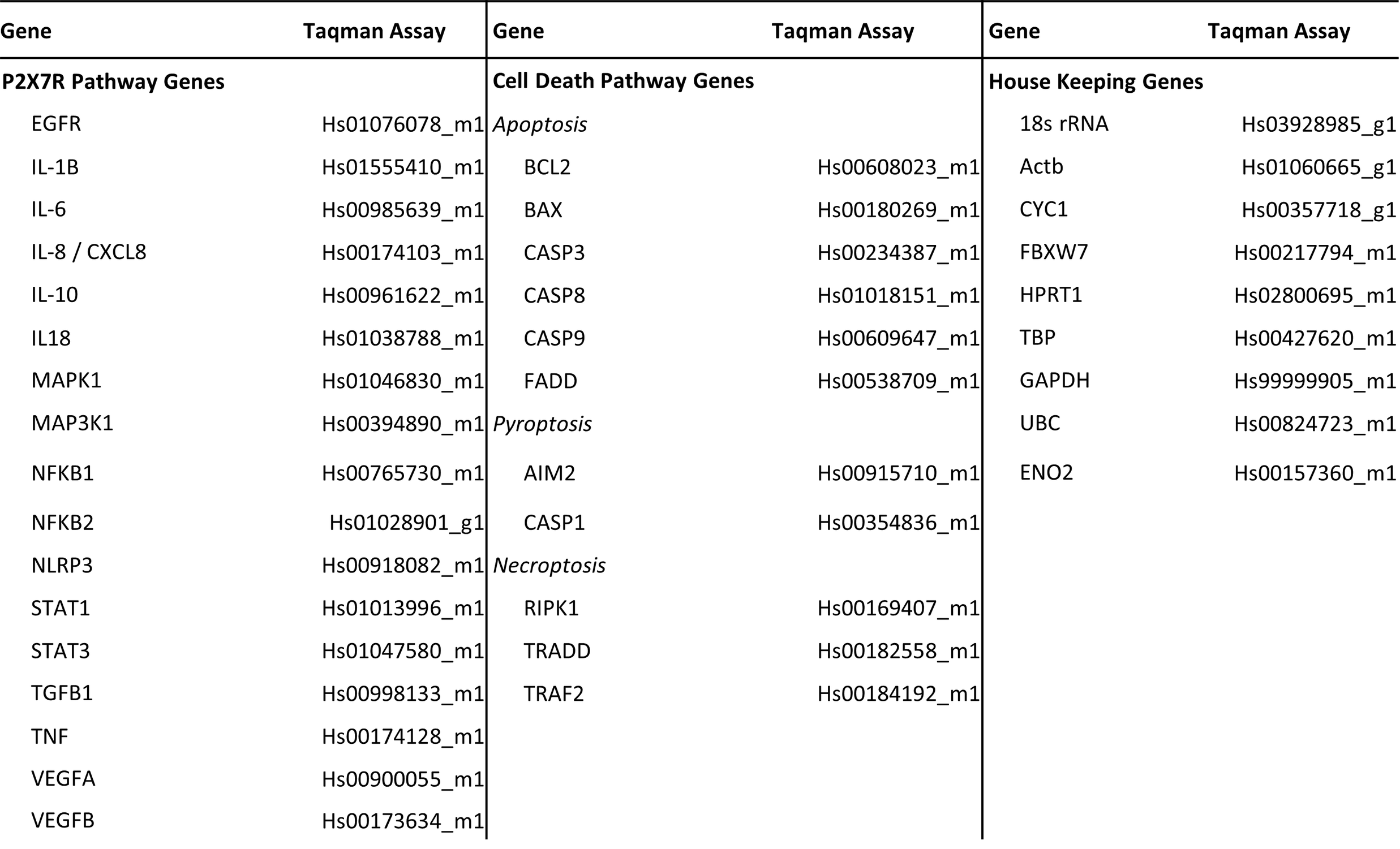

Supplement: S1 Table — (TIF) [file pone.0332212.s002.tif]
